# Known data on the therapeutic use of *Azadiracta indica* (neem) for type 2 diabetes mellitus

**DOI:** 10.6026/97320630018082

**Published:** 2022-02-28

**Authors:** U Vidhya Rekha, M Anita, S Bhuminathan, K Sadhana

**Affiliations:** 1 Department of Public Health Dentistry, Sree Balaji Dental College and Hospital, Pallikaranai, Chennai-600100, India; 2 Department of Prosthodontics, Sree Balaji Dental College and Hospital, Pallikaranai, Chennai - 600100, India

**Keywords:** *Azadiracta indica*, diabetes mellitus, antidiabetic activity

## Abstract

There has been growing interest for the therapeutic use of traditional herbs in the management of diabetes mellitus (DM) and its complications. Data shows the hypoglycemic activity of *Azadiracta indica* in diabetes. Therefore, it is of interest to document known data on the therapeutic use of *Azadiracta indica* (neem) for type 2 diabetes mellitus (T2DM).

## Background:

Herbal drugs are a great choice which is having more or less no side effect & adverse effects [1-2]. In recent years, there has been growing attention to alternative therapies and the therapeutic use of traditional herbs for the management of diabetes and its complications [3]. The plants present in our surroundings help to not only clean our environment but also its plant products are rich sources of antioxidants as well as contain phyto chemicals of medicinal uses 4]. Secondary metabolites often play an important role in plant defense against herbivores and other interspecies defenses. Humans use secondary metabolites as medicines, flavourings, pigments, and recreational drugs 5]. The Neem tree is an incredible plant that has been declared the Tree of the 21st century by the United Nations [6-7]. Neem oil is widely used as insecticides, lubricant, drugs for variety of diseases such as diabetes and tuberculosis [8,9]. It contains a wide variety of chemical constituents namely diterpenek (sugiol and nimbiol), triterpenes (beta-sitosterol and stigmasterol), terta-terpenoid (azadirachtin-k), sulphur containing compounds (nimbin, nimbidin, nimbinin and nimbidol), limonoids (maliantriol and nimbendiol), flavonol glycosides (nimaton, quercetin, myricetin, kaempferol) along with certain other compounds such as nimbolide, olichinolide B, 6-deacetyl-nimbin and azadiradione [10-12].

Diabetes mellitus (DM) is a diverse group of chronic metabolic disorder which can result from a defect in insulin secretion, insulin action, or both [13]. It is one of the chronic non-communicable diseases including the risk factor for vascular brain diseases, renal failure, blindness and diabetic wound [14]. The global burden of DM is enormous, with and estimated annual expenditure of 673 billion US dollars in 2015, which constituted 12% of global health spending for that year [15]. While in urban areas of low and middle-income countries, diabetes is well recognized as a public health priority; recent prevalence data suggest that diabetes is an increasing problem among rural populations as well [16]. The two main type of diabetes are type 1 diabetes and type 2 diabetes. Type 1 also known as insulin-dependent diabetes is characterized by insufficiency of the production of insulin as a result of damage of the beta cell of the pancreas [17]. Type 1 diabetic patients are likely to develop ketoacidosis. Type 2 diabetes known as non-insulin dependent diabetes is characterized by inefficient use of the insulin produced by the beta cells of the pancreas and it accounts for 90% cases [18]. Known data shows hypoglycemic activity of Azadirachta indica in diabetes [19] as the glucose level is significantly reduced by Azadirachta indica at 15th day in diabetic rat 20]. A report shows that chewing of Azadirachta indica leaves (Figure 2 - see PDF) in the morning for twenty-four days protected the body from diabetes [21]. Therefore, it is of interest to document known data on the therapeutic use of Azadiracta indica (neem) for type 2 diabetes mellitus (T2DM).

## Diabetes:

Ayurveda described Diabetes mellitus as condition of madhumeha [sugar loss in urine] or Ojameha [immunity and hormone loss] for treatment purpose is defined as the abnormality of carbohydrate metabolism which linked with low insulin [22]. Diabetes mellitus (Figure 1 - see PDF) is also defined as a metabolic disorder characterized by hyperglycaemia, hyper aminoacidemia and hyper insulinaemia which may also leads to decrease in the insulin secretions [23-check with author]. Glucose is a simple sugar that provides energy to all cells in the body. The cells take in glucose from the blood and break it down for energy; most of this is used for fuel. Glucose comes from the food you eat. Glucose gets absorbed from the intestines and distributed by the bloodstream to all cells. The body tries to maintain a constant glucose concentration in the blood. So, when over supply on glucose occurs, your bodystores the excess in the liver and muscles by making glycogen. When glucose is in short supply, the body makes glucose from stored glycogen or from the food that’s been eaten. To maintain a constant blood glucose level, the body relies on two hormones produced in the pancreas that have opposite actions: insulin and glucagon [24]. The pancreas plays a primary role of metabolism of glucose by producing and secreting the hormones like insulin and glucagon. The islets of langerhans produce and secrete insulin and glucagon directly into the blood. Insulin is a protein that is essential for proper regulation of glucose and for maintenance of proper glucose levels. [25] Glucagon is a hormone that opposes the action of insulin. It is secreted when blood glucose level falls. It increases blood glucose concentration, partly by stimulating the breaking down of stored glycogen in the liver by a pathway known as glycogenoysis Gluconeogenesis is the production of glucose in the liver from non-carbohydrate precursors such as amino acids [26-check with author]. Type 2 diabetes is often without symptoms in its early stages. That's the reason there are 40% of people with Type 2 diabetes are unaware of their disease. Whenthere are symptoms, they may occur gradually. If present, they usually are: feeling tired and weak, passing large volumes of urine especially during the night, having frequent infections, blurred eyesight, Weight-loss, Excessive hunger and thirst. Coma or death may occur as a result in Diabetic Ketoacidosis (caused by infection) and also People who smoke are a much higher risk at heart attacks, stroke, infections, and problems with poor circulation [24].

## Azadiracta indica (neem):

Azadirachta indica (neem), belonging to Meliaceae family native of Nepal, India, Bangladesh, Thailand and Pakistan [27]. The neem tree is an evergreen, or deciduous, fast-growing tree that can reach a height of 30 meters. The trunk is stout and the branches are wide and spreading, in severe drought may shed most or nearly all of its leaves. The flowers and fruits are borne in axillary clusters and when ripe the smooth ellipsoidal drupes are greenish yellow and comprise a sweet pulp enclosing a seeds consist of a shell with 1-3 kernels [28]. All parts of neem tree have been used traditionally as crude extracts in folk medicine, Ayurveda, Unani, Homeopathy, Chinese, several local systems within Africa and modern medicine to control diseases such as leprosy, malaria fever, smallpox, diarrhoea, cholera, intestinal helminthiasis and respiratory system [29-check with author, 30] and also in treatment of many infectious, metabolic or cancerous diseases [31,30].Numerous biological and pharmacological activities of this plant parts extracts have been studied and was reported to include antimalarial, antidiabetic, antibacterial, antifungal, anti-inflammatory, antioxidant, antiarthritic, antipyretic, hypoglycemic, antigastric ulcer, antiseptic, antiparalitic and antitumour activities [30-35].Furthermore, the oil extracted from the neem seeds has been reported to be effective as an antioxidant, insecticides, miticides, fungicide, nematocides and as an insect antifeedents and repellents [30,36]. More than 400 compounds have been isolated from different parts of neem tree including important bioactive secondary metabolites such as azadirachtin, nimbidin, nimbin, nimbolide, gedunin, non-isoprenoids such as flavonoids, alkaloids and steroids and many more [37]. Notwithstanding the chemical and biological virtuosity of neem, it has also been extensively explored for associated microorganisms, especially a class of mutualists called endophytic microorganisms (or endophytes) [38].

## Chemical Composition of Neem:

There are more than 300 compounds have been derived from diverse neem parts. The compounds have been broadly classified into two main classes which are isolated from neem: non-isoprenoids and isoprenoids. The compounds included in isoprenoids are diterpenoids and triterpenoids which contain limonoids, azadirone, protomeliacins, and its derivatives, vilasinin type of compounds, gedunin and its derivatives, Csecomeliacins such as salanin, nimbin, and azadirachtin. The compounds included in non-isoprenoids are carbohydrates (polysaccharides), proteins (amino acids), sulphurous compounds, polyphenolics such as glycosides and their flavonoids, dihydrochalcone, tannins, and coumarins, aliphatic compounds, etc. [37], margalonone, margalone, and isomargalonone [39].

## Traditional medicine:

The books such as AgathiyarGunavagadamBogar 7000, Agathiyarvaithyarathinasurukkam, Bogar 300 etc has numerous formulation of various parts of neem tree in medicine and it was transmitted from generations to generations by siddhars as Siddha system of medicine in Arabia, Persia, Turkey, China and other places get flourished.The medicinal properties of neem is depicted in 350 years old palm leaf manuscript conserved in the Centre for Traditional Medicine and Research (CTMR), Chennai, India which reveals the medicinal uses [40-check with author]. In India and Pakistan, the neem twigs are used for brushing or scrub the teeth. This practice may be one of the most effective and earliest forms of dental care.For the preparation of different medicines, all parts of neem tree (leaves, seeds, bark and flower) are used. Conventionally in India, patientgrief Chicken pox sleep on the leaves owing to its medicinal value [41]. This is a traditional treatment of Malaria used by Nigerians and other conditions is formed by the consumption of unspecified quantities without due regards to its toxicological effects. Aqueous extracts of seeds is used for head lies. Neem oil is good antiseptic for the treatment of skin as furuncles, eczema and intestinal worm infections [42].The pesticide activity has shown by neem and it is acknowledged for it. Against insects, one of the principal mode of action of neem is metamorphosis disruption and the bitter taste of neem keep the insects away from host plants and make it classic repellent and anti-feedent [43].A significant blood sugar lowering effects possessed by the alcoholic extract of neem leaves that are very beneficial against diabetes. Many fever-reducing and pain-relieving compounds are produced by neem that can assist in healing of cuts, earaches, burns, headache, sprains as well as fever. Neem extracts are as well used for the cure of malaria [44-check with author].

## Antidiabetic effect of Azadiracta indica (neem) leaf:

The longer the period of pre-treatment with the aqueous extract of A. indica leaves the better the protection against the onset of diabetes. The treatment of diabetic rats with aqueous extract of leaves of A. indica at a dose of 250 mg/kg body weight for 16 weeks resulted in gradual but significant fall in blood glucose and improvement in serum total, LDL and HDL cholesterol and triacylglycerol which increased in diabetic rats. It also showed improvement in body weight and reversed diabetic retinopathy. The mechanism behind it is not so clear but is said to be related to its antiserotonin activity [45].The ameliorative role of Azadiracta indica leaves extract against adverse effects of diabetes on the structure and function of the testicular tissue of rats neonatally induced by streptozotocin. The obtained results revealed significant decreased levels of testicular antioxidants, serum testesterone and significant increased levels of serum cholesterol, triglycerides, and low density lipoprotein but a remarkable decrease in high density lipoprotein in diabetic rats. Also, the testicular tissues of diabetic rats displayed several histological and ultrastructural changes especially in the spermatogenic and interstitial cells as well as overexpression of COX-2 protein if compared with control. Post-supplementation of neem leaves extract to STZ induced diabetic rats, revealed remarkable amelioration in most of disrupted estimated parameters [46].

Effect of oral treatment with combined leaf extract (CLE) of neem and bitter leaf on the prefrontal cortex of diabetic Wistar rats is known. Oral CLE produced normoglycemia in the treated hyperglycaemic rats. Besides, Nissl-stained prefrontal sections showed no morphologic deficits in all the groups except the untreated diabetic rats. In the latter, there was weak Nissl staining, while prefrontal MDA was significantly high at euthanasia, compared with control and CLE-treated rats (P>0.05). This study showed that untreated diabetes mellitus is associated with prefrontal Nissl body deficit and oxidative stress in Wistar rats. The absence of these deficits in CLE-treated rats suggests a neuroprotective effect of the extract in streptozotocin-induced diabetic rats. This may improve the cognitive function of the prefrontal cortex in diabetes mellitus. Administration of the extract can delay the onset of diabetes in diabetes prone subjects. This could be by quickening the sufficient production of insulin by the pancreas which activates the glucose transporters to transport glucose to the cells for effective utilization or possibly, the capability of the extract to regenerate the beta cells to produce enough insulin needed to signal the glucose transporters to carry glucose to the cells [47]. Azadiracta indica (neem) Plant hydroalcoholic extracts showed anti-hyperglycemic activity in streptozotocin treated rats and this effect is because of increase in glucose uptake and glycogen deposition in isolated rat hemi diaphragm [48,49]. The neem leaf extracts and seeds are used as an active ingredient as an effective cure for diabetes. It has been scientifically proven after a number of tests and research by leading medical institutes, that Neem parts have high efficacy in treating the disease. Natural Neem tablets are being manufactured and exported the world over for treating large number of patients. Neem leaf extracts improve the blood circulation by dilating the blood vessels and also helpful in reducing the need for hypoglycaemic drugs. Products made from Neem trees have been used in India for over two millennia for their medicinal properties. Neem products are believed by Siddha and Ayurvedic practitioners to be anthelmintic, antifungal, antidiabetic, antibacterial, antiviral, contraceptive and sedative. It is considered a major component in Siddha medicine, Ayurvedic and Unani medicine and is particularly prescribed for skin diseases. Neem oil is also used for healthy hair, to improve liver function, detoxify the blood, and balance blood sugar levels. Neem leaves have also been used to treat skin diseases like eczema, psoriasis, etc. However, benefits of Neem remain largely unknwon [50].

## Conclusion:

Parts of neem tree have been used traditionally as crude extracts in folk medicine, Ayurveda, Unani, Homeopathy, Chinese, several local systems within Africa and modern medicine to control diabetes, eczema and fever. Many scientific reports support the hypoglycemic activity of Azadirachta indica in diabetes is known. Numerous compounds have been isolated from different parts of neem tree. The bioactive secondary metabolites show the antidiabetic activity of Azadirachta indica. Thus, Azadirachta indica (Neem) is an alternative herb based medicine for the management diabetes mellitus.

## References

[1] Karimi A (2015). J Nephropharmacol..

[2] Efferth T, Koch E (2011). Curr Drug Targets..

[3] Gond AK, Gupta SK (2017). Int J Pharm Sci Invention..

[4] Zong A (2012). Carbohydrate Polymers..

[5] Pichersky E, Gang DR (2000). Trends Plant Sci..

[6] Choudhary DN (1990). Indian J Exp Biol..

[7] Kumar A, Navaratnam V (2013). Asian Pac J Trop Biomed..

[8] Kumar A (2009). Pak J Nutr..

[9] Tesfaye B (2018). Int. J. Eng. Tech. Mgmt. Res..

[10] Ghattopadhyay RR (1996). Gen. Pharmacal Col. Letrs..

[12] Pillai N, Santha KG (1981). Ind J Med Res..

[13] World Health Organization (2011). Diabetes Res Clin Pract..

[14] Chobanian AV (2003). JAMA..

[15] https://www.idf.org/.

[16] Hwang CK (2012). Diabetes Res Clin Pract..

[17] Afshin S, Muriel N (2006). Am J Ther..

[18] Spellman CW (2010). J. Am. Osteopath. Assoc..

[19] Khosla PS (2000). Indian J. Physiol. Pharmacol..

[20] Patil PR (2013). Natl J Physiol Pharm Pharmacol..

[21] Maragathavalli S (2012). Int. j. sci. nat..

[22] Gopala Krishna C (2018). Asian Pac. J. Health Sci..

[24] Rother KI (2007). N. Engl. J. Med..

[25] Karunanayake EH (1984). J. Ethnopharmacol..

[27] Abdullahi U (2020). Adv. J. Chem..

[28] Nisbet AJ (2000). Anais Socied. Entomol. Brasil..

[30] Hossain MA (2011). Pharmacognos. J..

[31] Kausar S (2017). J Parasit. Diseas..

[32] Paul R (2011). Cancer Biol. Therap..

[33] Ebong PE (2008). Am. J. Biochem. Biotechnol..

[34] Sultana B (2007). Food Chem..

[35] Hussain HE (2002). Indian J. Clin. Biochem..

[36] Ogbuewu IP (2011). Res. J. Med.Plant..

[37] Biswas K (2002). Cur. Sci..

[38] Ravindra N (2020). Planta Med..

[39] Dixit P (2015). World j. biol. med. sci..

[41] Maithani A (2011). J. Pharm. Res..

[42] Eid A (2017). Palest. med. pharm. j..

[43] Senthilkumar P (2018). J Pure Appl Microbiol..

[45] Ghattopadhyay RR (1996). Gen. Pharmacal Col. Letrs..

[46] Abd E-F (2020). Egypt.J. Basic Appl. Sci..

[47] Christian EO (2020). J. Phytopharm..

[48] Chattopadhyay RR (1987). Sch. Trop.med..

[49] Chattopadhyay RR (1987). Calcutta. Sch. Trop. Med..

[50] Ashish A (2020). Int J Ayurveda Pharma Res..

